# *Momordica charantia* L.: Functional Health Benefits and Uses in the Food Industry

**DOI:** 10.3390/plants14172642

**Published:** 2025-08-25

**Authors:** Lucian Vasile Bara, Ruben Budau, Alexandru Ioan Apahidean, Camelia Mihaela Bara, Carmen Violeta Iancu, Eugen Traian Jude, Gabriel Remus Cheregi, Adrian Vasile Timar, Mariana Florica Bei, Ionel Marius Osvat, Daniela Domocos

**Affiliations:** 1 Faculty of Environmental Protection, University of Oradea, 410048 Oradea, Romania; lbara@uoradea.ro (L.V.B.); rbudau@uoradea.ro (R.B.); cbara@uoradea.ro (C.M.B.); ciancu@uoradea.ro (C.V.I.); ejude@uoradea.ro (E.T.J.); atimar@uoradea.ro (A.V.T.); iosvat@uoradea.ro (I.M.O.); 2 Faculty of Horticulture and Business in Rural Development, University of Agricultural Sciences and Veterinary Medicine, 400372 Cluj-Napoca, Romania; alexandru.apahidean@usamvcluj.ro; 3 Faculty of Medicine and Pharmacy, University of Oradea, 410087 Oradea, Romania; ddomocos@uoradea.ro

**Keywords:** *Momordica charantia* L., nutritional value of bitter cucumbers, biochemical compounds, antihyperglycaemic properties, metabolic effects of *momordica*

## Abstract

Natural bioactive compounds found in *Momordica charantia* including polysaccharides, saponins, polyphenols, alkaloids, and notably polypeptide-p (often referred to as “plant insulin”)—have shown promising potential in shaping nutritional and therapeutic strategies for managing diabetes, metabolic disorders, and other nutrition-related diseases. Both retrospective and prospective analyses of bitter gourd’s functional properties such as its antioxidant, antitumor, immunomodulatory, and antibacterial effects highlight its innovative use as a food ingredient in developing targeted nutritional therapies. Assessing its applicability in the food industry, particularly through the fortification of products with bitter gourd powders, pulp, juice, or extracts, could enhance consumer acceptance and elevate the perceived quality of nutritionally superior foods. The nutrifunctional attributes revealed by its nutritional profile support the strategic integration of bitter gourd into various food formulations, contributing to a broader and more diverse range of dietary options. This diversification is especially valuable in addressing the dietary monotony often associated with diabetic nutrition plans, which continue to present significant challenges. The foundation laid by this review drawing on both theoretical insights and practical applications serves as a springboard for future research into the fortifying potential of bitter gourd-based preparations. Ultimately, such products may be recommended not only as nutritional supplements but also as part of clinical and hygienic-dietetic practices.

## 1. Introduction

*Momordica charantia* L. is cultivated on extensive areas in tropical and subtropical regions of the world, being widespread in India, China, Thailand, Japan, Singapore, Vietnam, the Amazon, East Africa, Brazil, China, Colombia, Cuba, Ghana, Haiti, India, Mexico, Malaya, New Zealand, Nicaragua, Panama, the Middle East, Central and South America [1,2]. It belongs to the Cucurbitaceae family and is valued for its bioactive compounds from the class of polysaccharides.

The name *Momordica* is linked to the serrated appearance of the leaf edges, which in Latin means “to bite” [1,3,4,5,6]. However, it is commonly known as bitter gourd, balsam pear, bitter melon [3], kugua, or karela [4], due to its intensely bitter taste [5], present in all parts of the plant.

Bitter gourd appears to be appreciated by consumers for its biologically active effects and distinctive bitter flavor, being regarded as a medicinal vegetable with significant pharmacological properties—both in its pulp and seeds—particularly in relation to diabetic conditions [6,7] and associated pathologies. Its hypoglycemic effect is primarily attributed to its antioxidant activity, which offers protective benefits to pancreatic β-cells. This antioxidant effect helps reduce glucose absorption in the intestines and glucose production in the liver, while enhancing glucose uptake by adipose and muscle tissues.

The hypoglycemic action is attributed not only to polypeptide-p but also to charantin and vicine, which have demonstrated insulin-like effects by reducing gluconeogenesis, increasing insulin production, promoting hepatic glycogen synthesis, and enhancing peripheral glucose oxidation [8]. Additional effects include inhibition of protein tyrosine phosphatase 1B (PTP1B), α-amylase activity [9], and 11β-hydroxysteroid dehydrogenase type 1 (11β-HSD1).

Although traditional medicine has used the leaves and even the roots of *M. charantia* to relieve toothaches treat diarrhea furunculosis and as antibacterial and antifungal agents [7,10], further studies are needed to confirm the efficacy and safety of its consumption.

The conceptualization of this review is linked to the importance of retrospectively analyzing the nutrifunctional properties of the species *M. charantia* L., identified through the nutritional profiling method.

As a prospective direction for evaluating the most optimal methods of food fortification, the nutritional profile provides guiding data regarding the expanded use of bitter gourd powder, extract, or pulp as fortifying agents. As a first stage in alternative medicine for the prevention and improvement of metabolic and nutritional pathologies, the nutritional profile is an extremely important indicator in assessing the impact of nutrifunctional compounds on reducing pathological status.

This study can offer real support to consumers affected by single or multiple conditions in diversifying the range of dietary products. Thus, preparations or extracts obtained from different parts of the plant can be recommended not only nutritionally as dietary supplements but also clinically, in hygienic-dietary practice.

The nutritional profile enables the innovation of new products aligned with dietary habits and traditions, with significant importance in formulating a global dietary plan by analyzing preventive benefits—both economically and scientifically.

Laczkó-Zöld et al. [2] claim that many preclinical and clinical studies conducted to date have shown protective effects of *M. charantia* L. against metabolic syndrome and its associated disorders.

Extracts derived from the fruit and seeds of bitter melon [2] have been shown to lower fasting glucose levels [11] and glycated hemoglobin A1c in type 2 diabetic animal models [9]. The aqueous leaf extract was shown to have an effect on obesity induced by a high-fat diet (HFD), through the regulation of lipid metabolism [12]. However, the limitations of the study regarding the pharmacological activities of *M. charantia* extracts are related to the fact that most research has been conducted on animals.

This review seeks to acknowledge and build upon an existing reality, directing research efforts toward the identification of functional nutrients in the species *M. charantia* L., with the objective of enhancing its practical use in the food industry. Achieving this goal involves the implementation of standardized, universally applicable methodologies that operate independently of conventional theoretical reference models. Such an approach may support the diversification of fortification techniques and strategies aligned with both theoretical and practical dietary frameworks, paving the way for the development of innovative food products.

Designing functional products with an innovative approach grounded in thorough planning, validation, and precise quality specification can lead to the production of high-standard foods, capable of delivering specific therapeutic effects for targeted pathologies.

This analysis is viewed as a systematic pursuit of excellence, offering data that can help meet consumer preferences while minimizing undesirable effects and enhancing utility in line with consumption specifications.

Most adverse effects associated with plant-derived products or ingredients, including herbal supplements, can be attributed either to poor quality or to processing and contamination of the final product. Good manufacturing practices are among the most critical tools for ensuring the quality of fortified food products, pharmaceuticals, and herbal medicines.

A thorough and critical review of existing scientific literature is essential, particularly regarding the safety of incorporating *M. charantia*-based products as functional ingredients in a variety of dietary foods—such as baked goods, pastries, dairy items, juices, teas, salads, and others.

Thanks to its synergistic interaction with other plant-derived components, fortifying select food products with bitter gourd—whether in the form of powder, pulp, juice, or extract sourced from the whole fruit—at a dosage of 5 g per serving may minimize adverse effects without compromising the intended functional efficacy.

## 2. Methodology

**Figure 1:**
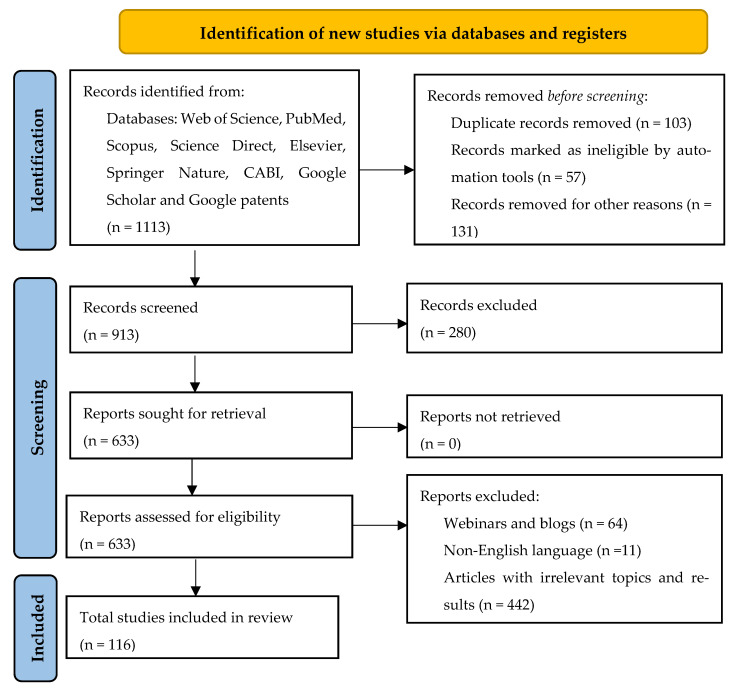
PRISMA 2020 flow diagram for the present review.

In selecting the bibliographic data concerning the biochemical composition and nutrifunctional effects of *M. charantia* L., we utilized the PRISMA 2020 flow diagram [13]. The search engines employed included Web of Science, PubMed, Scopus, Science Direct, Elsevier, Springer Nature, CABI, Google Scholar, and Google Patents, adhering to the Preferred Reporting Items for Systematic Reviews and Meta-Analyses (PRISMA) guidelines [13]. The selection criteria and steps, along with the number of studies incorporated into our analysis, illustrated in Figure 1, were derived from the most current data obtained from recent research publications, encompassing both in vivo and in vitro studies published within the last seven years.

The keywords included in the search were the following: *M. charantia* L., bioactive compound of *momordica*, antioxidant capacity/activity of *M. charantia*, hypoglycaemic effect of *momordica*, antihyperglycemic properties, and nutritional value of bitter melon.

Research examining the nutritional impact of *M. charantia* L., which has been published in languages apart from English and Romanian, has been excluded. Furthermore, we omitted studies that are over 10 years old and those lacking relevance or deviating from our specific focus. A thorough selection procedure was conducted, resulting in a total of 118 studies included in this review from an initial identification of 1113 studies (Figure 1).

## 3. The Bioactive and Nutrifunctional Effects of *Momordica charantia* L. on Health

**Figure 2:**
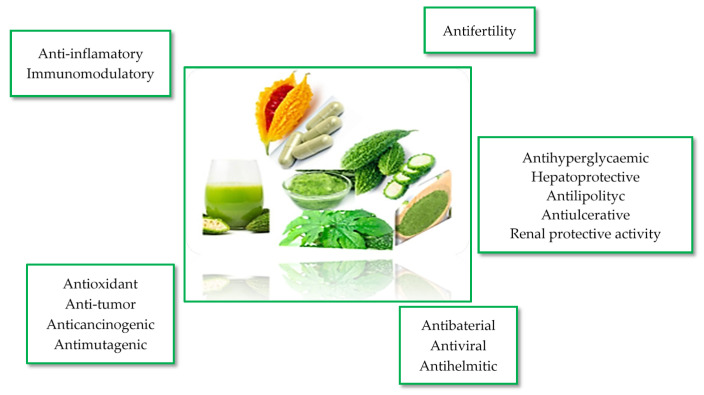
The effect of bioactive compounds identified in *M. charantia* L., [1,6,7,10].

The nutrifunctional effects and bioactivity of *M. charantia* L. species have been evinced by scientific studies identified based on a retrospective analysis of research. The identified studies have reported the diverse effects of *M. charantia* L. on different metabolic mechanisms (Figure 2), emphasizing its potential as an alternative remedy, particularly for the treatment of diabetes [3,10,14].

The functional health benefits and the applicability in the food industry of the constituent parts of the *M. charantia* L. species can be retrospectively supported by a number of studies. In this regard, studies investigating the efficacy and safety of *M. charantia* L. preparations on the reduction in elevated plasma glucose levels in patients with prediabetes and type 2 diabetes mellitus have led to the observation of improved glycemic control [2,15,16].

The present observations are informed by data from prior investigations [2,3,10,11,12,15,16] evaluating the therapeutic potential of monoherbal adjuvant formulations derived from *M. charantia* L. Nevertheless, a number of systematic reviews [3,10,11] underscore the necessity for further rigorous studies before such preparations can be recommended for clinical application, primarily due to reported adverse effects.

In the majority of studies included by Laczkó-Zöld et al. [2] in their meta-analysis on the antidiabetic effects of *M. charantia* (bitter gourd) extracts, no severe adverse events were reported. Mild side effects such as headache and gastrointestinal discomfort—including episodes of diarrhea and epigastric pain were noted approximately one month following administration [17]. Consumption of bitter melon pulp at a dosage of 6 g/day was associated with an increased incidence of diarrhea, occasionally accompanied by flatulence, nausea, and constipation. Furthermore, after 12 weeks of continuous administration of encapsulated momordicin powder, certain diabetic patients exhibited adverse reactions, including anorexia with nausea, diarrhea, foamy urine, and skin rash [17]. The cytotoxic and anti-inflammatory properties of cucurbitane-type triterpenoids isolated from *M. charantia* shoots and leaves were evaluated by Chou et al. [18]. The study revealed that momordicin I exhibited cytotoxic effects on normal cells at concentrations exceeding 10 µM. In contrast, momordicin II demonstrated comparatively lower cytotoxicity, while momordicin IV and 3β,7β,25-trihydroxycucurbita-5,23-dien-19-al (TCD) showed no significant adverse impact on cell viability across a concentration range of 0.1–100 µM. These results suggest that the presence of potentially toxic constituents warrants cautious consideration in the use of *Momordica* shoots and leaves for therapeutic purposes.

The results of animal studies [19] show that *M. charantia* seed extract, at LD_50_ values of 50 μg/mL, can have lethal effects on the embryo, and at sublethal doses it manifests potential negative effects on the blood system and reproductive health, inducing multiple anomalies. The dose of 5 μg/mL of seed extract did not induce developmental delays in embryos, but cardiac hypertrophy was evident. The fruit extract proved to be harmless, with no significance regarding the mortality of embryos treated with doses up to 200 μg/mL [19]. However, it was observed that at doses ≥30 μg/mL crude fruit extract, embryos developed cardiac hypertrophy, an aspect that was not observed at doses < 30 μg/mL. Following these findings, it is particularly important to note that the teratogenicity of the seed extract and the toxicity of the fruit extract of *M. charantia* on the cardiac system induce warnings regarding the use of fruit extracts and seeds of *M. charantia* by pregnant patients diagnosed with diabetes mellitus to reduce the risk of heart rhythm disorders and the risk of spontaneous abortion [19].

Therefore, it is recommended to avoid the use of products obtained from *M. charantia* in pregnant or breastfeeding women due to the high risk of fetal malformations, manifested especially in the myocardium by blocking transcription factors normally required for myoblast specification, thus preventing the formation of specialized striated muscle fibers to ensure rhythmic muscle contraction [20]. To be recommended for pregnant women [19], pharmacological studies should be conducted to determine the dose and duration of treatment in order to reduce health risks. In adults, cardiac toxicity has not been observed, except for one study in which mild atrial fibrillation was observed after consuming a large amount of *M. charantia* juice [21] due to the different absorption of liquid forms compared to administration as a supplement. Ethanol extract of *M. charantia* seeds tested in animals showed a significant impact on spermatogenesis, inducing histological changes in both testes and accessory reproductive organs. In females, aqueous leaf extracts decreased plasma progesterone and estrogen levels in a dose-dependent manner [22].

Due to the fact that *momordica* seeds contain compounds similar to vicines, they could induce a form of hemolytic anemia known as favism, manifested as glucose-6-phosphate dehydrogenase (G6PD) deficiency, which is why there is a warning regarding consumption in this case, thus reducing the risk of health disorders [19,23].

Excessive consumption of *M. charantia* juice [24], due to the content of lectin, a potentially toxic compound identified in the peel and seeds of *M. charantia*, can inhibit protein synthesis at the intestinal level, an effect that may be related to the separation of Vicia faba glycosides present in the seeds.

Consumption of 500 mL of concentrated *M. charantia* juice [25] can cause acute gastric ulcers and intestinal bleeding as effects of charantin, lectin [26], and some potentially toxic alkaloids.

In vitro studies on *M. charantia* L. proteins, specifically α- and β-momorcharin, have demonstrated their inhibitory effect on the HIV virus. In order to combat infections, its extract can also be used as a broad-spectrum antibacterial agent [27,28]. These advantageous effects are ascribed to the substantial concentrations of phytoconstituents that have been utilized for millennia to treat a wide range of illnesses [1,6,7,10].

A retrospective analysis on the content in nutrifunctional elements and the bioactive effects of *M. charantia* L allowed the identification of a rich content of triterpenoids [3,7], saponins recognized as triterpenoids and cucurbitacins, polypeptides [29], flavonoids, alkaloids, and sterols [1], as well as marcronutrients: carbohydrates, proteins, lipids, and others [30]. All these are considered useful tools in the treatment of several components of MetSin before starting pharmacologic therapy or as supplements to medical treatment [2].

Scientific studies on specific human body disorders have demonstrated the significance of *M. charantia* L. (MCP), a polysaccharide, as one of the most important constituents of the plant, with antioxidant [31,32], antibacterial, antitumor [33], and immune regulatory [34] properties. These qualities are just a few of its many roles and effects. Additionally, according to Liu et al. [35], it may be considered potential medication to treat diabetes and pathologies associated to it.

Liu et al. [35] focused on *M. charantia* L. polysaccharide (MCP) and its impact on diabetic retinopathy (DR) and diabetes mellitus (DM). They have also demonstrated its effects in improving systemic indicators such as body weight, general health, inflammation, and retinal tissue apoptosis [36]. The inflammatory factor IL-6, which is produced by a variety of cells, including periacinal fibroblasts, under the influence of TNFα (tumor necrosis factor), was assessed by the authors in order to illustrate the activity of MCP. IL-6 is also the primary mediator in the synthesis of acute phase proteins, such as MCP-1 (monocyte chemoattractant protein), in response to inflammatory cytokines. By controlling the actions of cytokines, MCP can further regulate inflammation and apoptosis of retinal neurons and micro vessels, strengthening their anti-inflammatory and anti-apoptotic properties. According to Liu et al. [35], MCP may therefore be a new bioactive medication for the prevention and treatment of diabetic retinopathy as well as a selective medication for the prevention and amelioration of DM.

Regardless of whether it is used for prophylaxis or treatment, *M. charantia* L., which is thought to be a novel source for the treatment of diabetes, shows good antidiabetic and antioxidant activities and holds increased potential in the treatment of diabetes [4].

Among the bioactive compounds isolated from *M. charantia*, the peptide BG-4 has demonstrated notable anti-inflammatory properties. BG-4 inhibits the production of interleukins IL-6 and IL-8, as well as tumor necrosis factor-α (TNF-α), in lipopolysaccharide (LPS)-activated macrophages [36]. This activity is associated with reduced nuclear translocation of the p65 subunit of nuclear factor kappa B (NF-κB), a pivotal regulator of immune and inflammatory responses [37]. Additionally, the anti-inflammatory action of the bioactive peptide BG-4 has been shown to downregulate anti-apoptotic Bcl-2 protein expression while upregulating the pro-apoptotic protein Bax under chronic inflammatory conditions [38].

Momordicoside K, kaempferol, and quercetin—bioactive compounds identified in *M. charantia*—exhibit notable therapeutic potential, as evidenced by pharmacological studies linking them to mechanisms involving BRCA-suppressor genes. These genes play a critical role in DNA repair and the preservation of genomic stability, thereby mitigating the accumulation of high-risk mutations that contribute to tumorigenesis [39]. The elucidation of these molecular pathways offers valuable insight into the anticancer properties of bitter gourd and highlights its promising role in the therapeutic management of breast cancer [40]. This potential is further supported by extensive preclinical evaluations, which consistently demonstrate the favorable profile of *M. charantia* in this pathology.

Clinical interventions based on laboratory determinations have demonstrated that bitter gourd is certainly a plant that deserves to remain relevant for future research as a fortifying ingredient in some foods, thus aiming to complement or improve conventional approaches to chemotherapy and radiotherapy [41]. These characteristics may transform compounds with functional potential into promising agents for increasing therapeutic efficacy and simultaneously minimizing adverse effects, representing a key element in the research of therapeutic options in oncology [42,43].

Major compounds that have been isolated from fruits, seeds, peels, and leaves of *M. charantia* L. (Table 1), as hypoglycemic agents include charantin, charin, cryptoxanthin, cucurbitin, cucurbitacin [44,45], vicin, polypeptide-β or β-insulin, lanosterol, lauric acid, linoleic acid, rosmarinic acid, rubixanthin, trehalose, trypsin inhibitors, zeaxanthin, lutein [46,47,48] and kakra compounds. To these, additional substances such as polypeptides, triterpenoids, saponins, flavonoids, alkaloids, and sterols may be added [49]. These have been demonstrated to have positive effects on overall health as well as on particular metabolic disorders. Blood glucose regulation is thought to be related [50] to charantin (a mixture of steroidal saponins), β-polypeptide, vicin, and momordin [49] analogues (e.g., momordinol, momordicillin, momorcharin, and momordicin).

P-polypeptide is an insulin-like protein, which is also called P-insulin or v-insulin [51]. Charantin, momordenol, and momordicillin are important active compounds that possess insulin-like structure and chemical properties [52]. Momordicin II and cuguaglycoside G could also stimulate insulin secretion.

The typical hypoglycemic mechanisms of the species *M.a charantia* L. are mainly attributed to proteins/peptides, polysaccharides, phenolic compounds, triterpenoids, alkaloids, and charantins (steroidal saponins [53,54,55], identified in this plant (Table 1). An important role in these mechanisms is manifested by the modulation processes of the intestinal microbiota, which are essential for hypoglycemic and antihyperlipidemic activities. Thus, the identification of potential mechanisms whereby *M. charantia* L. improves insulin sensitivity could represent adjuvant nutrifunctional mechanisms in stimulating resistance to insulin in diabetes and related pathologies, such as obesity and dyslipidemias [56]. The therapeutic potential in combating obesity has led to the observation that *M. charantia* could represent an extremely promising ingredient for the development of products and supplements aimed at supporting weight management.

The fruit juice of *M. charantia* L. has multiple influences on glucose and lipid metabolism, strongly counteracting the harmful effects of high-fat diets. In addition, antioxidant and anti-inflammatory activities contribute greatly to its anti-hyperglycemic properties [18,56]. Long-term oral administration of extracts from the fruits of *M. charantia* L., in adequate doses, may be beneficial in improving diabetes [57].

**Table 1:** Content of biofunctional compounds identified in fruits, seeds, leaves, and pericarp of *Momordica charantia* L.

Plant Parts		Category	Type	Subtype	References
Functional bioactive components 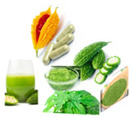	Fruits 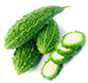	Phenolic compounds	Flavonoids Non-flavonoids	Catechin, Epicatechin Quinic acid Quercetin Rutin Kaenferol Isorhamnetin Gallic acid, Gentisic acid, Chlorogenic acid, Tannic acid, Protocatechuic acid, Vanillic acid, Syringic acid, P-coumaric acid, Benzoic acid, Sinapinic acid, O-coumaric acid, t-cinnamic acid, t-ferulic acid, Tannins, Luteolin-7-O-glycoside, Apigenin-7-O-glycoside, Caffeic acid, Naringenin-7-O-glycoside	[18,32] [32,58,59]
		Carotenoids	Lutein, α and β Carotene, Zeaxanthin, β Cryptoxanthin, Lycopene		[59,60]
		Cucurbitacine Triterpenoids	Charantin, Cucurbitacins, Kuguacins A-S, Momordicine I, II, III, Momordicoside D Karavilagenin A-E, Saponins, Goyasaponins sapogenins	Triterpenoid glycosides Diosgenin	[2,32,45,49]
		Phytosterols	Decortinone, Clerosterol, Ergosterol Peroxide, Stigmasterol, Campesterol, β sitosterol β-sitosterol 3-O-β-D-glucoside 5,22-stigmasterol 3-O-β-D-glucoside		[32,60]
		Alkaloids and polypeptides	Polypeptide-P, Lectin α-momorcharin, β-momorcharin, γ-momorcharin, δ-momorcharin, ε-momorcharin, Peroxidase		[22,61,62], [27,52,54,55]
	Seeds 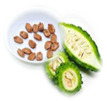	Phenolic compounds	Flavonoids Non-Flavonoids	Catechin, Epicatechin Kaenferol Rutin, Vanillin, Quercetin, Luteolin, Chlorogenic acid, P-coumaric acid, Ferulic acid, Caffeic acid, Gallic acid, Salicylic acid, Syringic acid, Ethyl gallate, Naringenin, Apigenin, Pyrogallol	[1,22,49,63]
		Carotenoids	Clorofile, β-carotene Lycopene		[64]
		Cucurbitacine Terpenoids	Cucurbitan, Saponins, Triterpenoids	- - Charantin, Momordicine I, II, III, Karavilagenin A-E, Kuguacins A-S	[22,59,62]
		Phytosterols	Glucosides	β-sitosterol, Campesterol, Stigmasterol, Stearic acid, α-linoleic acid, α-eleostearic acid	[1,63]
		Alkaloids and polypeptides	Polypeptide-P Polypeptide K Lectin		[1,60,65]
	Leaves 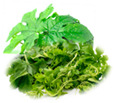	Phenolic compounds	Flavonoids Non-flavonoids	Kaempferol 3-glucuronide, Kaenferol-3′,4′-methylenedioxy-5,7-dimethylepicatechin, Kaenferol 7-arabinoside, Orientin 7,3′-dimethyl ether, Quercitin, Luteolin, Limocitrin 3-rhamnoside, Tannins, Hydroferulic acid, Chlorogenic acid fragments, Abscisic acid fragments, Myriantic acid, Madasiatic acid, Vernolic acid, N-Cinnamide, Apigenin	[12]
		Carotenoids	Clorofil, β-carotene		[22]
		Cucurbitacine Terpenoids	Diterpenoid Cucurbitacins Saponins	Rosmaricine Charantin, Cucurbitacin alkyl, Oleanane, Ursane Momordicine I, II, III, Momordicinin, Momordicin I, II, Momordin B, Momordicoside F1, F2, Momordicoside K, Phytolaccasaponin G	[12,22,62]
		Phytosterols	Glucosides Phytolaccoside E	Goyaglycoside b,c, Apigenin 6-C-arabinosyl-8-C-glucoside,	[12]
		Alkaloids			
		Anthraquinones			
	Peel 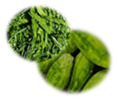	Phenolic compounds	Isoflavones, Ddihydroflavones, Flavanols, Flavonoids, Chalcones, Flavonoid Carbonosides Dihydroflavonols	Eriodictyol-3′-O-glucoside, Eriodictyol-7-O-(6′-acetyl) glucoside, Eriodictyol-8-C-glucoside, Eriodictyol-5,3′-Di-O-glucoside, Pinostrobin, Naringin, Liquiritigenin-4′-O-glucoside (liquiritin) Herbacetin, Isolhamnetin-3,7-O-diglucoside, kaempferol-3-O-glucoside-7-O-rhamnoside Dihydro kaempferol-7-O-glucoside, Quercetin-3-O-(6″-acetyl), galactoside, Kaempferol-3-O-neohesperidoside, Isorhamnetin-3-O-(6″-acetylglucoside, Kaempferol-3-O-(6″-malonyl) glucoside, Quercetin-3-O-(6″-malonyl) galactoside, Quercetin-7-O-(6″-malonyl) glucoside, Sexangularetin-3-O-glucoside-7-O-rhamnoside Kaempferol-4′-O-glucoside, Aromadendrin-7-O-glucoside, Tricin-7-O-glucoside, Limocitrin-3-O-arabinoside, Limocitrin-7-O-glucoside, Saponarin-4′-O-glucoside, Sakuranin, Kaempferol-6,8-di-C-glucoside Isorhamnetin-3-O-rutinoside (narcissin) - Vitexin, Isovitexin, Isoorientin Orientin-7-O-glucoside, Vitexin-2″-O-rhamnoside Hhesperetin-5-O-glucoside, Hhesperetin-7-O-(6″-malonyl) glucoside	[12,66]
		Tanin	3-O-methylgallic acid, 1-O-galloyl-rhamnose, 1-O-galloyl-D-glucose.		[12,66]

In a comparative metabolic study [66] on the quantitative detection of flavonoids in the pericarp of three *momordica* cultivars, dark green, light green, and white, 90 flavonoid substances were identified, which included 3 isoflavone subtypes, 9 subtypes of dihydroflavones, 7 subtypes of flavanols, 34 subtypes of flavonols, 26 subtypes of flavonoids, 4 subtypes of chalcone, 5 subtypes of carboxyl-flavonoids and 2 subtypes of dihydroflavonols, and three types of tannin, 3-O-methylgallic acid, 1-O-galoyl-ramnose and 1-O-galoyl-D-glucose. Varying amounts of vitexin and isovitexin flavonoids were additionally recognized, dependent on color intensity.

The comprehensive examination of secondary metabolism and antioxidant capacity aimed at uncovering metabolite accumulation patterns in various cultivars of *M. charantia* L. [59] resulted in the identification and documentation of 27 distinct metabolites. Among these, there are 15 phenolic acids, 2 flavones, 1 flavanone, 3 organic acids, 1 coumarin, and 1 lignin. The phenolic compounds exhibit a range of activities, including antioxidant, insecticidal, antiparasitic, anti-inflammatory, antibacterial, antidiabetic, cicatrizant, antidiuretic, cytotoxic [67], and antitumor effects [51,52]. Additional research focused on isolating unknown active principles from bitter gourd may uncover a novel source of anti-inflammatory and anticancer medications [68].

Meanwhile, flavonoids, which are secondary metabolites of plants, are crucial for numerous biological functions, and the majority of flavonoids are produced downstream of phenolic acids [69,70]. This study identified 42 flavonols, 21 flavones, 4 isoflavones, and 2 flavanones across seven varieties of bitter gourd. HPLC analysis [49] demonstrated that all fractions of bitter gourd contained bioactive compounds, with naringenin and catechin being most abundant in the fruit, while epicatechin, ferulic acid, kaempferol, and p-coumaric acid were found in higher concentrations in the pericarp. Additionally, chlorogenic acid, caffeic acid, gallic acid, and apigenin were most concentrated in the seeds. When compared to the fruit and pericarp, the seeds exhibited a greater concentration of bioactive components that have significant functional effects.

Sedentary lifestyles and high energy intake from food consumed throughout the day are two of the many factors that are thought to promote obesity and increase the incidence of metabolic syndrome and diabetes [56]. Drug therapy, specifically designed for diabetes, is undoubtedly beneficial, but the side effects associated with drug therapies lead to increased interest in alternative traditional therapies with proven effects [71]. The role of diet and dietary interventions is highlighted in numerous scientific studies, and the role of plants and plant-based products is of increasing importance [72]. A low GI diet can be attained by consuming foods that are high in amino acids, essential fatty acids, phytonutrients, and phytochemical compounds.

In an attempt to develop a balanced diet plan from the perspectives of macronutrients and micronutrients, foods are combined, depending on their respective nutritional profiles. This allows for the correction of nutritional deficiencies based on the nutritional quality indices of each individual food.

## 4. The Nutritional Profile of *Momordica charantia* L. and Its Applications in Food Industry

The nutritional profile facilitates the development of novel products that are simultaneously associated with customs and eating habits, allowing each product to have a significant place in the global food diet. Regardless of a consumer’s current condition or degree of impairment, researchers aim to improve the nutritional value of foods that significantly affect their health based on the food’s nutritional profile [69,72]. As poor food choices and unhealthy meals are the primary causes of non-communicable diseases, the real objective is to promote consumer health through informed food choices.

Nutrient profiling (NP) is a technique for categorizing and assessing foods based on their nutrient content [73,74,75,76]. Employing the NP model to evaluate different food items proves beneficial in guiding food selections, assisting consumers in making informed decisions about healthy alternatives. [77]. Currently, NP models are utilized to enhance public health awareness and to mitigate non-communicable diseases (NCDs). Over 20 nutrient profile models are implemented on nutrition profile websites, health recommendation platforms, consumer guides, dietary guidelines, nutrition labeling, and/or health claims associated with the labeling of packaged or pre-packaged food items, in compliance with the European Union Regulation No. 1169/2011 [77].

Determining the nutritional profile of the species *M. charantaia* L., based on the retrospective identification of biochemical elements and nutrients that demonstrate curative potential, is the first step in establishing nutrition as a modern and effective medicine [4]. Such an affordable and promising nutritional treatment option may represent a secondary alternative to traditional medical treatments due to its efficacy, limited side effects, and availability of nutritional components.

By conducting additional research on bioactive components found in food, contemporary medicine can broaden its treatment strategies for metabolic disorders, including T2DM and associated conditions [4].

**Figure 3:**
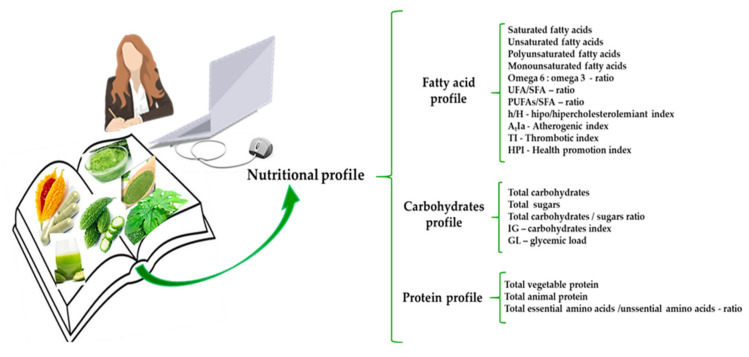
The nutritional profile of the food can support informed food choices.

The nutritional profile, which has been prospectively linked to a lower risk of developing MetSin (metabolic syndrome), can help the population make informed dietary decisions regarding individual needs. (Figure 3).

The insights derived from specialized literature have resulted in the conclusion that, for consumers with specific dietary requirements, it is essential to develop their nutritional profile as a summary based on the macro- and micronutrient content present in *M. charantia* L.

Therefore, especially in certain pathological conditions, the values established based on the nutritional indices used in this study may serve as acceptance points for the consumption of food products derived from *M. charantia* L. The nutritional value of a food product attests to its quality and serves as a key factor in evaluating the quality of food meant for human consumption. This is because food products with higher nutritional values provide the body with nutrients that are more easily absorbed (Figure 3).

Nutritional profiling models are developed and analyzed in order to assess the nutritional value and quality of food, as well as its calorie content and quantity of macro- and micronutrients. Furthermore, the nutritional inconsistencies resulting from inaccurate information on the product label are explained because they could create false perceptions (Figure 3).

Comparing and explaining the nutritional content of each food is one approach to reduce the likelihood of misunderstandings. This can be done by stating the energy, macronutrient, and micronutrient content that is correlated with reference values (%CR) that are pertinent to the general public (Figure 3).

According to studies by Abo Taleb et al. [78], the presence of substantial amounts of proteins, fats, and carbohydrates resulted in an energy content estimate that ranged from 235.12 kcal/100 g to 311.80 kcal/100 g. The protein content identified ranged between 13.21 g/100 g DW and 27.88 g/100 g DW; the amount of fat identified was between 5.2 g/100 g DW and 6.11 g/100 g DW, and the amount of carbohydrates was between 32.34 g/100 g DW and 34.31 g/100 g DW. Knowing the energy value of food does not require the study of metabolic pathways but rather the quantitative assessment of food energy that will be used by the body.

To judiciously assess the nutrifunctional quality of *M. charantia* fruits, nutritional profile indices were determined based on existing literature [1,54,70,79,80,81] to emphasize the most sensitive nutrifunctional properties. These indices potentially facilitate the novel identification of incorporating *momordica* products, both in their fresh, unprocessed form and as a fortifying agent in specific products. This incorporation is intended for inclusion in the dietary plans of consumers with related diabetic and metabolic conditions, ultimately contributing to the development of effective nutrient therapies.

### 4.1. Protein Profiles

The amount of protein identified in several studies [1,49,59,72,81,82], with the aim of determining the protein content in products obtained from *M. charantia* L., showed significant values in terms of both essential and non-essential amino acid content. The non-essential amino acids identified [80] were (aspartic acid, serine, glutamic acid, alanine, g-aminobutyric, pipecolic acid, glycine, and proline, in the amount of 395.3 mg/g protein in fruit and 381.8 mg/g protein in seeds, respectively [1,59,80].

Among the 8 essential amino acids, 7 have been recognized: valine, leucine, isoleucine, methionine, lysine, phenylalanine, threonine, and tryptophan. Furthermore, in addition to these 7 essential amino acids, the semi-essential amino acids tyrosine and cysteine, along with the relatively essential amino acids arginine and histidine, were also identified [1,52,80], in an average amount of 532 mg/g protein, in fruit and 485 mg/g protein in seeds, respectively.

The decrease in insulin secretion deficiency, which is a protein-like hormone, along with disturbances in insulin deficiency primarily observed in target tissues such as muscle, adipose tissue, and liver, can also lead to the autoimmune destruction of pancreatic β-cells (adult autoimmune type 1 diabetes), as noted by Bogun et al. [83]. This process may also be influenced by the protein content found in *momordica*.

In light of this finding, enhancing certain food items with *momordica* may alleviate the restrictive impact of specific essential amino acids, thereby improving the irregularities in protein metabolism, which, alongside lipid and carbohydrate metabolism, contributes to the intricate process that leads to the onset of diabetes mellitus [84,85].

Increased plasma insulin concentrations facilitate the use of amino acids for the synthesis of muscle proteins, thereby promoting the incorporation of amino acids such as lysine, which is important for the growth and development of living organisms, as well as tryptophan and arginine [86]. Additionally, phenylalanine serves as a precursor in the biosynthesis of para-coumaric acid, with further conversion resulting in the creation of caffeic acid and ferulic acid [59].

In the diet of diabetic individuals, protein and lipids will compensate for the energy shortfall resulting from carbohydrate intake recommendations, which are approximately 40–45% lower than those for non-diabetics. Consequently, with a more consistent intake of protein and lipids, the stores of tissue proteins and lipids will be preserved.

The amino acid quantities identified made it possible to determine the essential AA/non-essential AA ratio, yielding informative values of 1,35 for the fruit and 1,46 for the seeds, respectively, values that give the product a high functional level in terms of protein content. The role of essential amino acids in the functionality of vital organs is helping to increase consumer interest in the consumption of *momordica* fruit, both as a salad and in the form of various *momordica*-based products.

### 4.2. Fatty Acid Profile

Based on the analysis of the identified fatty acid composition, it was found [27,87] that the lowest amount of palmitic acid (3.20–5.29%) was recorded, with linoleic acid coming in second (4.81–6.98%). However, the amounts of oleic acid (15.26–16.01%), stearic acid (20.21–24.20%), and eleostearic acid were significantly higher than those of palmitic and lyoleic acid. Eleosteraic acid is a conjugated fatty acid, (C18:3-n5), identified in the seeds of *M. charantia* L. in a proportion of 42.66–61.99%, with important functional properties through the increased ability to inhibit some cancer cells [27,34].

The composition of fatty acids, phospholipids, and phytosterols in the fruits of *M. charantia* L as evaluated by standard analytical methods [80], facilitates the setting of a detailed lipid profile, thereby enabling a nutritional assessment of bitter melon fruits. According to the data obtained [81], the fatty acid concentrations in *M. charantia* fruits were arranged as follows, in ascending order: linolenic acid (2.38) < stearic acid (7.52) < oleic acid (20.18) < palmitic acid (23.64) < linoleic acid (45.47).

Arachidonic, arachidic, palmitoleic, margaric, behenic, erucic, lignoceric, myristic, lauric, capric, and caprylic acids were detected in minimal quantities, not exceeding 1.0% [80]. Furthermore, the findings indicated a low level of monosaturated fatty acids (MUFA) at 20.41%, while the values for polyunsaturated fatty acids (PUFA) were recorded at 2.44% [27]. The phospholipid composition revealed that phosphatidylcholine had the highest concentration at 100.31 mg/100 g fat, whereas lysophosphatidylcholine and phosphatidic acid exhibited the lowest concentrations at 12.62 mg/100 g fat. The levels of phytosterols were generally low, with the exception of sitosterol, which had a value of 153.28 mg/100 g fat [80].

The available information regarding fatty acid composition [34,76,78] facilitated an analysis of the nutritional lipid profile. During the lipid profile assessment, the Atherogenic Index (AtIa), Thrombotic Index (IT), and Health-Promoting Index (HPI) were evaluated, as they are frequently utilized for their considerable effects on metabolic disturbances, effectively demonstrating the nutrifunctional benefits to health.

Moreover, the antidiabetic, cardioprotective, and nutrifunctional properties of *momordica* fruit corroborated by AtIa [87] are attributed to the absence of proinflammatory fatty acids such as myristic acid (C14:0) and lauric acid (C12:0). Aremu et al. [80] reported that the level of palmitic acid (C16:0) resulted in an Atherogenic Index (AtIa) [87] of 0.34, indicating an anti-inflammatory impact that is beneficial for cardiometabolic, diabetic, and nutrition-related conditions [87].

AtIa = (12:0 + 4 × 14:0 + 16:0)/(Σ MUFA + Σ PUFA)(1)

The dietary atherogenic index is considered optimal at values of 0.38–0.39. Thus, the greater the value of this index, the less pronounced the nutrifunctional effect will be.

The correlation between fatty acids and human health, supported by IT, is recognized as one of the nutritional indices that offers protective effects against diabetes, cardiovascular diseases, and related metabolic disorders, as evidenced by numerous studies assessing the degree of thrombogenicity [88].

The TI of *momordica* fruits is recorded at an average value of 0.78, which is derived from the fatty acid composition (myristic C14:0, palmitic C16:0, and stearic C18:0) in relation to the unsaturated fatty acid content, as noted by Aremu et al. [80] and calculated using the formula established by Ulbricht and Southgate [87].

TI = (C14:0 + C16:0 + C18:0)/[0.5 × ΣMUFA + 0.5 × Σ(n − 6) + 3 × Σ(n − 3) + Σ(n − 3)/Σ(n − 6)] (2)

Lower TI values suggest a higher nutritional quality of the food under examination, and its consumption may lower the risk of coronary heart disease (CHD), metabolic and nutritional disorders, as well as diabetic foot gangrene. The HPI additionally emphasizes evaluating the nutritional value of dietary fats and the impact of fatty acids on cardiovascular (CVD) and related metabolic health through the following formula [89]:

HPI = ΣMUFA + ΣPUFA/[C12:0 + (4 × C14:0) + C16:0] (3)

According to this formula, HPI is the inverse of AtIa, and it is currently used in research on the nutritional quality of food products, and the values of this index range from 0.16 to 0.68 [89], compared to the values of AtIa, which is considered optimal at values of 0.38–0.39. Determination of HPI in *momordica* fruits based on fatty acid composition [80] supports the nutrifunctional capacity of this species, with an HPI of 2.89. The higher the values of this index, the higher the health benefit of the product [88].

Nutritional profile indices, due to renal, coronary, and body weight complications associated with type 2 diabetes mellitus (T2DM2), supported by abnormalities in lipid, protein, and carbohydrate metabolism [89], are predictors of the nutrifunctional quality of *M. charantia* products.

Nutritional studies on the impact of food and diet on lipid, carbohydrate, and protein metabolism imbalances associated with type 2 diabetes show that elevated levels of free fatty acids in the liver, skeletal muscle, and pancreas can produce persistent lipotoxicity [90].

It is our belief that the determination of these nutritional profile indices is essential for the design and approval of a new food product that possesses genuine functional properties.

### 4.3. Carbohydrate Profile

GI and GL are important indicators of carbohydrate profile, decisive in assessing the epidemiological risk of diet-induced impairment of glucose metabolism [91]. GI and GL represent the physiological basis for the prioritization of foods according to their effect on postprandial glycemia, with important implications for pathologies sensitive to macro- and micronutrient intake, particularly in diabetics and those with associated cardiovascular and metabolic diseases.

Their weight in the assessment of the carbohydrate profile has led to the consideration that these indicators should be included in the food quality assessment of products intended for human consumption due to the GI-lowering capacity of the fortified food. This aspect is due to the low GI of *momordica* fruit; thus, a carbohydrate-rich food consumed as part of a meal will undergo changes in the GI of the meal according to the average of all GI values of the component foods of the meal.

The carbohydrate content of *M. charantia* L. fruits identified in the literature [1,34,52,78,79,80,92,93,94] ranged from 32.34 g/100 g DW to 34.31 g/100 g DW, showing a GI = 15. This low GI value is based on the available carbohydrate content, but may change depending on processing and preparation methods, physico-chemical characteristics such as acidity or starch type and content [92], and the presence of protein, fat, and fiber [59,92,93].

It is also acknowledged that these indicators ought to be conveyed to the general public and healthcare professionals via food guides or specific GI and GL databases for each country, food composition tables [91], and nutritional labeling of foods [77], which would reach consumers’ tables to facilitate informed food choices.

In this manner, GI would aid in comprehending the body’s physiological responses to foods that contain carbohydrates [92].

The glycemic load index (GL) is determined by taking the quantity in grams of carbohydrates, multiplying it by the glycemic index, and subsequently dividing the result by 100, as per the following formula:

GL = (IG xportion of carbohydrates in the food, expressed in g)/100(4)

The quantity of carbohydrates [78] and the glycemic index (GI) facilitated the calculation of glycemic load (GL), which yielded values of 4.85 and 5.14. Since these values are under 10, *momordica* is classified within the low glycemic load food category. The advised daily carbohydrate consumption should be less than 40 GL. Foods with a GL of 20 or higher are categorized as having a high glycemic load.

### 4.4. PRAL Score

Using the PRAL score, the antiulcer effect was examined in relation to the micronutrient content [78,86]. It allowed for the nutritional-statistical assessment of renal acid load with negative potential, as well as the evaluation of the acid-base effect on the body after consumption of *M. charantia* L. [95,96]. The PRAL concept was developed more than 20 years ago by Remer and Manz, and it has a physiological foundation [95,97].

The calculation of PRAL score takes into account dietary protein (g), phosphorus, potassium, calcium, and magnesium (mg), using the following formula [95,96,98]:

Potential renal acid load (PRAL) = 0.4888 × dietary protein (g) + 0.0366 × dietary phosphorus (mg) − 0.0205 × dietary potassium (mg) − 0.0125 × calcium (mg) − 0.0263 × magnesium (mg)(5)

Dietary acid-base load is actually a balance between acidogenic foods, which are protein-containing foods, and alkaligenic foods fruits and vegetables that provide basic precursors [96]. Alkaline supplementation can overcome starvation-induced acidosis, thereby leading to greater weight loss and better physical performance [99]. Thus, a food product with a negative PRAL has alkaline properties superior to acidic ones.

Mineral elements, identified in *momordica* fruits, were P in the amount of 70–140 mg/100 g DW, K in the amount of 8–170 mg/100 g DW, Ca in amount of 20–50 mg/100 g DW, and Mg 16 mg/100 g DW. Other mineral elements identified were Fe in the amount of 2.2–9.4 mg/100 g DW, Na in the amount of 3–40 mg/100 g DW, Zn in the amount of 0.1 mg/100 g DW, Mn in the amount of 0.08–0.32 mg/100 g DW, and Cu in the amount of 0.18–5 mg/100 g DW [4,79,100,101].

By using the PRAL score, it was found to have negative values, ranging from 2.53 to 1.96. Therefore, the lower the PRAL score is, respectively, more negative, the higher the food’s alkaline capacity [96].

In terms of carbohydrate content, identified amounts of 32.34 g/100 g DW–34.31 g/100 g DW [1,101], which would provide 12.43–13, 20% of DZR.

The data evaluated and presented in this review indicate that, as regards health concerns, the seeds of *M. charantia* L. are a good source of vitamins and minerals. Of all the minerals found in the seeds of *M. charantia* L., calcium had the highest concentration, ranging from 440.96 μg/g DW to 1728 μg/g DW [1,86,100]. Phosphorus ranged from 134.65 to 142.39 μg/g DW, copper from 2.85 to 3.52 μg/g DW, iron from 41.10 to 45.03 μg/g DW, zinc from 12.41 to 13.47 μg/g DW, and Na from 2200 μg/g DW. In contrast to other authors, Abo Taleb et al. [75] found substantially higher amounts of macroelements; for example, Ca was found in amounts of 511.98 mg/100 g DW, Mg in amounts of 427.21 mg/100 g DW, K in amounts of 1439.22 mg/100 g DW, Mn 0.79 mg/100 g DW, Fe 4.32 mg/100 g DW, and Zn in amounts of 1.37 g/100 g DW. This can supply 64% of the RDA for Ca, 85.44% of the RDA for Mg, 41.12% of the RDA for K, 43.2% of the RDA for Fe, and 9.13% of the RDA for Zn [100].

### 4.5. Real Nutritional Factor (FNR) and Biological Value (VB)

The nutritional quality of plant-origin food products is given by the index of biological value and the real nutrient factor (NRF), determined on the basis of the nutrients they transfer to the human body through the process of metabolism.

The nutritional biological value of the species *M. charantia* L., assessed in relation to the key nutrients it offers and quantitatively determined using the formula known as the “real nutrient factor” (NRF, expressed per 100 g of product), which was introduced by RINNO in 1965, has resulted in average values of 43.39 [1,82,100,101]. The more positive these values are, the greater the nutrifunctional impact of food on health.

NRF = Vit. C (mg)/20 + carotenoids (mg) + fibers (g) + Ca (mg)/100 + Fe (mg)/2(6)

In the research focused on the creation and implementation of a novel index for evaluating the nutritional quality of plant-based foods, the role of protein was examined alongside fiber, vitamins A and C, calcium, and iron in comparison to the formula provided by RINNO. Other genuine nutrient factor evaluation formulas, which investigated the benefits of incorporating additional minerals and vitamins [102,103,104], included:

V1: NRF6.3—protein, fiber, vitamins A, vitamins C, and the minerals calcium and iron.
V2: NRF8.3—Protein, fiber, vitamins A, C and minerals Ca, Fe, Mg, K
V3: NRF11. 3—Protein, fiber, vitamins A, C, E, B12 and minerals Ca, Fe, Mg, K, Zn
V4: NRF15.3—Protein, fiber, monounsaturated fatty acid, vitamins A, C, D, E, B12, B1, B2, B9, and minerals Ca, Fe, K, Zn

NRFn.3 indices (where n = 6–15) are derived from unweighted [105,106], averaged sums presented as daily percentage values (VZ) for essential health nutrients (n) and for nutrients advised to be consumed in restricted quantities (LIM) (n = 3). The nutrients that should be restricted consist of saturated fat, added sugar, and sodium.

The formula proposed by Bielka, 1965, can be used to determine the biological value of vegetables (BV) taking into account the protein content.

The biological value of *M. charantia* fruits obtained mean values of 166.2, which assigns it high functional values [1,83,101,102].

The nutritional profile indices and their analysis serve primarily as tools to guide consumers regarding the nutritional quality of food, but they also provide information that needs to be clinically validated [103,104,105,106] through careful monitoring of participants in nutritional studies who have a low risk of exacerbating their current pathological condition.

The establishment of the degree of applicability in the food industry is based on the premises of this review, which aimed to identify the starting point of new research on the fortification of foods using powder, paste, or concentrated extract of *M. charantia* L. However, this requires a degree of development, particularization, and loading with meanings so as to be a pathfinder for the continuation and not the cessation of research, but through an evolution that can prevent the occurrence of associative surprises.

The development of the premises was focused on those aspects that state the diversity of the theoretical and practical orientations of the research and that require the possible synthesis in a methodological project appropriate to the level of theoretical development on the nutrient content of the species *M. charantia* L., with proven functional effect.

The validation of data regarding the applicability of the species *M. charantia* L. in the food industry is based on the degree of functional impairment of some human metabolic processes, according to the requirements of scientific research; however, aiming to comply with some scientific criteria, namely:

- objectivity: in order to carry out an objective study, the retrospective results presented by different authors on the identification of functional nutrients were rigorously compared;
- fidelity: in order to give the results a high degree of fidelity and identify the most optimal fortification method, the data obtained were repeatedly analyzed;
- validity and comparability: the retrospective results on the content of nutrients with increased functional potential respected the established criteria, and in order to ensure the repeatability and verification of the data presented retrospectively, an attempt was made for the evaluation rules to be clearly and objectively presented in such a way as to allow the establishment of the most optimal experimental design on the fortification, with *Momordica charantia* L., of several food groups.

### 4.6. Current and Future Applications of M. charantia as a Functional Ingredient

The validation of retrospective data on the content of nutrients with functional properties opens new research directions related to the sustainability of the *M. charantia* L. species within the food industry, particularly in light of consumer demands. Our interest in developing novel food products with *M. charantia* L. as the primary ingredient in varying concentrations stemmed from the fact that this plant has been increasingly investigated or even used in the production of different foods or supplements. We shall analyze the following products, in which *M. charantia* L. has been added as an ingredient in the form of tea, syrup, paste, or powder: oat biscuits, rye/oatmeal cakes, savory pastries, jams, jellies, syrup, soft drinks, such as lemonade, yogurt, ice cream, or other food formulas of the salad type.

Our interest in conceptualizing new products based on *M. charantia* is linked to the excellent nutrifunctional properties of this plant, based on the various preparations previously obtained, dating back to 2005.

The development of an innovative processing method that reduces the risk of excessive consumption but maximizes the activities of the functional components of *M. charantia* presents a necessity of the current population regarding dietary diversification.

Thus, in an attempt to obtain a new yogurt formula, [107] used *M. charantia* juice in a ratio of 3:7 with fresh milk as raw materials in which *Lactobacillus bulgaricus* and *Streptococcus thermophilus* were used as lactic cultures to stimulate fermentation in a ratio of 1:1. The fortified yogurt exhibited a distinct flavor profile attributable to the added ingredient, characterized by a noticeably sweeter and more sour taste compared to conventional plain yogurt. This product, considered promotional, presented an increased importance as a new functional food due to both yogurt and bitter lemon having numerous benefits for public health [107].

*M. charantia* powder can replace 4% of the flour used in pasta production [108], leading to products with increased nutritional properties compared to plain pasta. In this case, the mass fraction of *M. charantia* was 9%, and the starch mass fraction was 12%. The resulting pasta had a saturated-to-unsaturated fat ratio of 1:4 and also presented lower production costs [109].

Using *M. charantia* powder, Liu et al. [110] obtained the best formulation for effervescent tablets, with good sensory properties and a unique bitter taste that was easily accepted by consumers. The effervescent tablets were made from a mixture containing 4% *M. charantia*, 17.8% sucrose, 3.6% sweeteners, 31.1% sodium bicarbonate, 6.7% citric acid, and 35.6% maltodextrin.

As a raw material, *M. charantia*, together with zinc gluconate as a color protector and β-cyclodextrin as a bitterness-masking agent, was used to produce *M. charantia* granules [111].

As a nutritious beverage with reduced sugar content, *M. charantia* juice has been used in various concentrations and combinations with other food products. Wang et al. [112] developed a new drink based on bitter gourd and peanuts as raw materials, using xylitol as a sweetener.

A mixture of 30% *M. charantia* juice with 15% Campanulaceae extract, to which 8% sugar was added as a sweetener and 0.1% citric acid as a color stabilizer, resulted in a beverage appreciated by consumers as nutritious [112]. It was reported to have effects on gastric ulcers and chronic atrophic gastritis, as well as a tonifying effect on the lungs and spleen.

To obtain beverages with significant nutritional properties, *M. charantia* juice was combined with apricot juice in a 2:7 ratio [113,114]. Another nutritious drink was formulated using 10% *M. charantia* juice, 20% citrus juice, 7% sugar, 3% honey, and 0.2% citric acid [115]

Using fresh milk and *M. charantia* juice in an 8:2 ratio, with the addition of 9% sugar and 0.15% compound stabilizer, a milkshake-type preparation was obtained [116].

In 2014, an individual in China was granted an invention patent (CN103610180A) for a beverage containing the following raw materials: 40–60 parts bitter gourd juice, 10–30 parts carrot juice, 20–40 parts sorb juice, 10–30 parts yellow peach juice, 1–5 parts salt, and 0.01–0.1 parts stabilizing agent.

The data presented in this review serve as motivation for us to continue research into the applicability of this species in the food industry, aiming to develop new products similar to those described in the literature but designed based on the nutritional profile evaluation of each ingredient used. This approach seeks to achieve superior quality and enable the establishment of appropriate dietary recommendations.

## 5. Conclusions and Perspectives

Nutritional profile indices offer a means to assess the potential nutrifunctional and medicinal properties of *M. charantia*. However, the data derived from such evaluations require rigorous testing once the species is incorporated as a fortifying agent in food products.

To draw conclusions about its health-related nutrifunctional effects, fortified food must be examined through a systematic and multifaceted research approach, with careful interpretation of nutritional profile results.

The nutritional profile (NP) in health recommendation systems serves as the main indicator in dietary guidelines, ensuring the accuracy of nutritional health claims applied to product labels.

In the field of quality systems, NP represents a highly important factor of designed quality, through which individual nutritional values are expressed at a set level by comparing multiple variants that play a key role in defect prevention.

At the clinical level, recommending the therapeutic use of foods enriched with *M. charantia* necessitates consideration of medical, biological, and pharmacological factors, particularly regarding efficacy and in vivo bioavailability.

In this regard, the concentration at which the powder, paste, tea, and juice can be used as functional ingredients in pastry products, yogurt-type dairy products, ice cream, lemonade, or salad must be carefully analyzed both in terms of positive and negative health effects in order to establish recommendations for the consumption of these products.

The retrospective analysis of the interactions between the various forms of presentation and use of products derived from *M. charantia* L. as ingredients with fortifying properties represents a significant and integral part of the concept of functional food, aimed at improving consumers’ quality of life by reducing the impact of diabetic and associated metabolic pathologies.

The prospective analysis revealed that the growing interest in consuming biofunctional foods is largely driven by concerns over the adverse effects of conventional pharmaceutical treatments. However, it is often overlooked that certain food products may contain antinutritional compounds or may be suitable only for specific categories of consumers.

This observation serves as a crucial starting point for further research focused on identifying the most effective method of integrating *M. charantia* L. into the dietary regimen of individuals with diabetic pathologies, with the view of developing efficient nutritional therapies.

Due to the increased susceptibility to food-related diseases in the otherwise healthy population, the modern population, where eating behavior disorders can be identified, is at risk for metabolic and nutritional conditions.

## Data Availability

Not applicable.
